# Self-healing in leprosy: A systematic review

**DOI:** 10.1371/journal.pntd.0012434

**Published:** 2024-09-12

**Authors:** Sophie C. W. Stuetzle, Ann-Kristin Bonkass, Wim H. van Brakel, Anne Schoenmakers, Anil Fastenau

**Affiliations:** 1 Department of Global Health, Institute of Public Health and Nursing Research, University of Bremen, Bremen, Germany; 2 Marie Adelaide Leprosy Center, Karachi, Pakistan; 3 evaplan GmbH at the University Clinic Heidelberg, Heidelberg, Germany; 4 NLR international, Amsterdam, Netherlands; 5 Erasmus MC, University Medical Center Rotterdam, Rotterdam, Netherlands; 6 German Leprosy and Tuberculosis Relief Association (DAHW), Wuerzburg, Germany; 7 Department of Health, Ethics & Society, Care and Public Health Research Institute CAPHRI, Faculty of Health, Medicine and Life Sciences, Maastricht University, Maastricht, Netherlands; University of Calgary, CANADA

## Abstract

**Background:**

Leprosy, caused by *Mycobacterium leprae*, affects multiple body systems and can lead to preventable disability if untreated. While multidrug therapy (MDT) has been available since 1982, historical evidence suggests that untreated leprosy can resolve spontaneously. Nevertheless, the prevalence of self-healing worldwide, as well as factors determining self-healing, remain unclear.

**Methods:**

A systematic review was conducted in 2023 with data from PubMed, Infolep, and Web of Sciences data bases, along with a google search. Data extraction and analysis followed PRISMA guidelines and were summarized in a separate Excel sheet. Included were English-language studies on self-healing in leprosy, regardless the year of publication.

**Results:**

We included six studies spanning from 1938 to 1978 exploring the incidence of self-healing in different countries and continents. Children, paucibacillary (PB) cases, and possibly males showed a higher probability of self-healing with an average healing time of two years.

**Discussion/Conclusion:**

Recent research on self-healing in leprosy is scarce and evidence limited. This is primarily due to ethical concerns regarding withholding effective treatment of diagnosed patients, and because of the absence of an agreed definition of self-healing. Nevertheless, self-healing appears to be a plausible phenomenon influenced by geographic and demographic factors, and the type of leprosy. We recommend further research on self-healing in leprosy, as it provides insight into the human immune system and the determinants of this phenomenon. More insight could help adapt clinical practices and public health strategies, thereby contributing to an effective management and control of this disease.

## Author summary

Leprosy, a chronic infectious disease caused by Mycobacterium leprae, presents significant health challenges worldwide, with the potential for avoidable disabilities if left untreated. While MDT has substantially reduced the disease burden since its introduction in 1982, historical records suggest that some cases of leprosy exhibit spontaneous resolution without medical intervention. However, the prevalence of self-healing globally and the factors influencing this phenomenon remain unclear.

The review identified six studies conducted between 1938 and 1978, which examined the occurrence of self-healing in various countries. These studies revealed that children and individuals with PB leprosy demonstrated a higher likelihood of self-healing, typically within an average period of two years. Despite the limited recent evidence due to ethical considerations, self-healing appears to be a plausible phenomenon influenced by patient-specific characteristics and the type of leprosy infection.

Ultimately, this paper presents a compelling case for future research on self-healing in leprosy, as it provides an interesting view on the human immune system as well as the determinants of this phenomenon. Our recommendations emphasize the necessity of such investigations to adapt clinical practices and public health strategies, thereby contributing to an effective management and control of this disease.

## Introduction

Leprosy is a chronic infectious disease caused by *Mycobacterium leprae (M*. *leprae)* that affects peripheral nerves, skin, upper respiratory tract mucosa and eyes [1]. If the disease is treated too late, nerve damage can lead to preventable disability [2]. The incubation period of leprosy is relatively long and can vary widely, ranging from several months to more than twenty years [3]. Besides the slow incubation period of clinical manifestations, the variation in clinical presentation—also dependent on the immune response of the patient—contributes to the major challenges in early case detection, contact tracing, and interruption of the transmission of the disease. Since 1982, the World Health Organization (WHO) has recommended multidrug therapy (MDT) as the standard treatment for leprosy. MDT is provided free of charge by WHO to countries reporting new leprosy patients since 1995 [4,5].

MDT has transformed the treatment of leprosy leading to a reduced reported prevalence worldwide; people are no longer defined as “leprosy patients” after finishing MDT. However, evidence from the pre-treatment era suggests that certain cases of leprosy may also self-heal if left untreated [6]. The true global incidence of self-healing among leprosy cases is unknown, since it is not possible to determine how many people are infected. The reasons why only some leprosy patients may self-heal while others develop symptoms and further clinical manifestations are not fully understood. Furthermore, since effective treatment is available, it is not ethical to conduct longitudinal randomized studies to explore the phenomenon of leprosy self-healing in humans. Despite older studies indicating that leprosy shows a tendency to self-heal, no uniform definition has been established [6,7,8,9,10]. Research has solely suggested that spontaneous recovery from leprosy signs and symptoms can be the result of a slow and gradual development of immunity to the bacteria causing leprosy [9]. Furthermore, a study by Martinez et al. [11] has shown that Polymerase Chain Reaction (PCR) testing revealed high rates of exposure to *M*. *leprae* in certain populations. This study found a significant number of individuals testing positive for the presence of *M*. *leprae* in hotspot areas, suggesting infection [11]. However, despite high PCR and Phenolic Glycolipid 1 (PGL-1)—positivity rates, the actual reported incidence of individuals developing overt clinical symptoms of leprosy is much lower than the percentage of positive PCR results [11,12]. The meta-analysis of Penna et al. [12] to identify the presence of Immunoglobulin M (IgM) antibodies against the species-specific PGL-1 through enzyme-linked immunosorbent assay (ELISA) showed that the sensitivity of PGL-1 as a predictor of clinical leprosy varied from 2 to 39%. This suggests that a substantial proportion of individuals exposed to *M*. *leprae* do not progress to overt disease or may remain asymptomatic [12]. The difference between PCR and PGL-1 seropositivity rates and the reported incidence of leprosy supports the notion that a significant proportion of individuals indeed experiences resolving of the infection, preventing the progression to the clinical disease state [13,9]. Along with that, past studies have suggested the probability of self-healing to be associated with factors such as age, sex, type of infection, host’s immune response related to genetic factors or close contact in households and families, although a causal association is not confirmed [13].

Despite that self-healing or spontaneous regression in leprosy cases has been described by many researchers over the years [7,8,9,10], evidence remains limited. During the COVID-19 pandemic however, the incidence of leprosy cases decreased by a large percentage [14]. Between 2019 and 2020 there was a decline of 37% in new case detection (from 202,185 to 127,396) due to preventive measures related to the COVID-19 pandemic [14]. The restrictions of the COVID-19 pandemic significantly hindered global leprosy control measures such as active case finding, case detection, treatment, and prophylaxis [15]. Health facilities faced challenges in diagnosing and treating new leprosy patients and those with reactions, as they were overloaded by the pandemic. The supply of leprosy medication became challenging amid the disruptions caused by the ongoing health crisis [15]. While an impact of the pandemic on the new case detection of leprosy was therefore expected [16], it is noteworthy that some countries (e.g. Afghanistan and Bolivia) have not yet documented an increase in cases to the level before COVID-19, nor have the “missed cases” in other countries been accounted for up to the end of 2022 [14]. Although several factors might have contributed to this observation (e.g., ongoing health care providing backlogs, political changes, or effects secondary to low endemicity), one factor may be self-healing of leprosy cases over the course of the pandemic. Moreover, mathematical models have included self-healing when predicting trends in new case detection, thus emphasizing its possible relevance [17,18].

The aim of this systematic literature review is to provide more understanding of the role of self-healing in leprosy and to reveal existing knowledge gaps regarding this topic. Understanding the factors associated with self-healing could potentially shed light on novel control strategies and aid in the quest for leprosy elimination.

## Methodology

### Search strategy

A systematic review was conducted from June 2023 to September 2023 in the databases PubMed, Web of Science and Infolep by two assessors. The review aimed to examine evidence on self-healing for leprosy cases globally. The following search terms consisting of controlled vocabulary for PubMed (MeSH), “leprosy” as well as free text terms being “Hansen disease”, “spontaneous”, “heal”, “self heal” and “remission” were used in the search strategy. The search terms were connected by the Boolean Operators “AND“, “OR“. Furthermore, by applying the snowball principle, additional literature was identified in the list of references of selected studies and via a Google search. The overall search was conducted by performing a Title (TI) and Abstract (AB) search (S1 Table). The overall review follows the methodology of the PRISMA checklist [19].

### Inclusion and exclusion criteria

Only studies that matched pre-set eligibility criteria were included. Papers needed to address the issue of self-healing of those who were diagnosed with leprosy. We put no restriction on the publication year of potential studies, as there were limited papers available that assessed the occurrence of spontaneous self-healing in leprosy patients. The review was restricted to English literature. Articles that were not accessible or did not contain any epidemiological or other quantitative data were excluded. Furthermore, it is worth noting that included studies were conducted within ethical bounds, as there was no treatment available for leprosy at the time of the study.

### Data extraction and analysis

**Fig 1:**
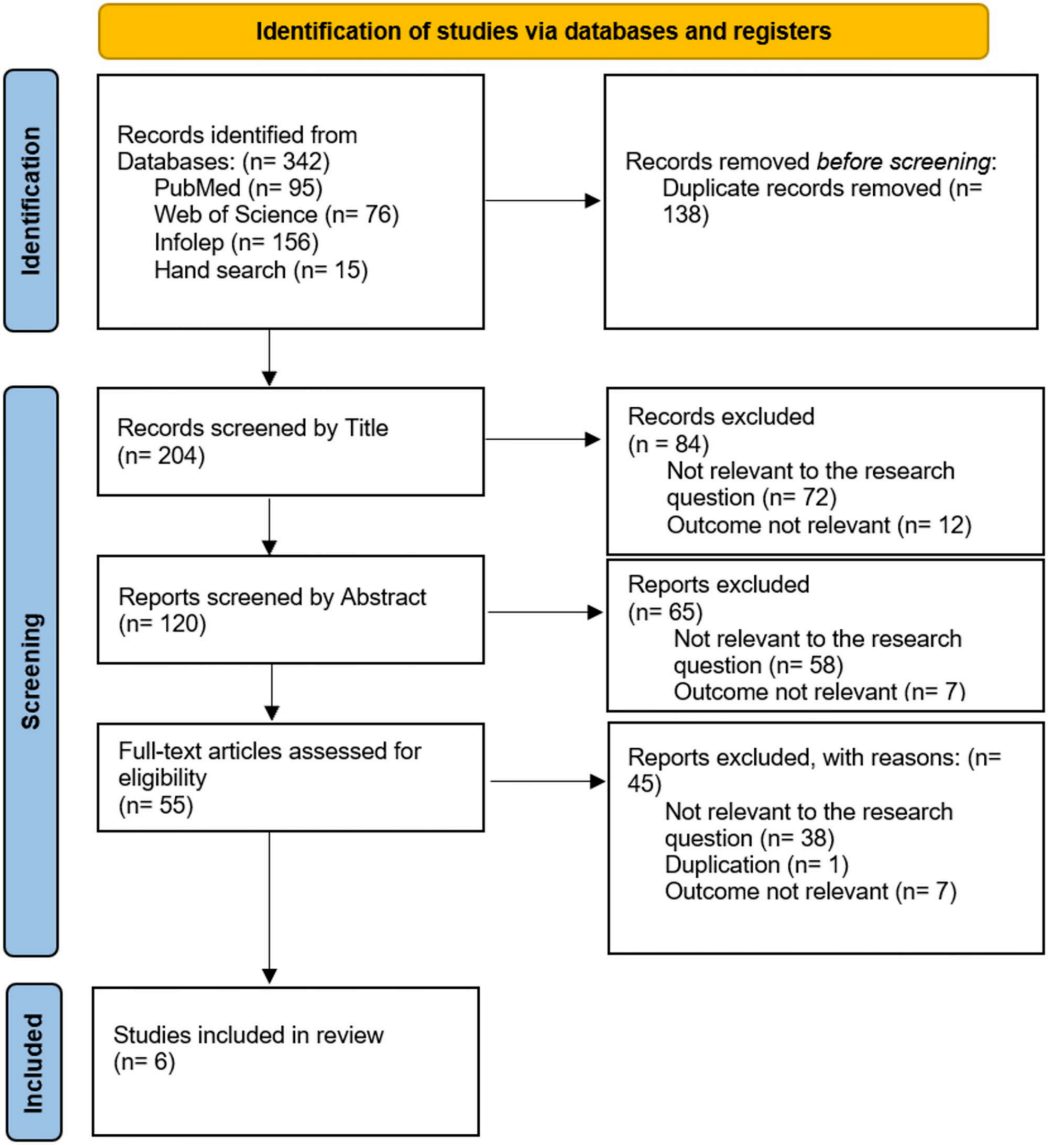
Flowchart of included studies.

The data extraction was done independently, whereby all researchers were involved. The found articles in the databases were deduplicated. Afterwards, articles were screened for eligibility, first by title, then by abstract and finally using full text (Fig 1). The data of the resulting six articles were extracted using Microsoft Excel. The developed spreadsheet included the title of the paper, author, and publication year as well as how the papers answered the set research question (S2 Table). A comprehensive analysis was conducted, differentiating between different themes of leprosy-healing in the found literature, such as demographic factors, country and incidence of self-healing. The analysis followed the methodology of the PRISMA guidelines [19].

## Results

The initial literature search yielded 342 studies in three databases. Duplicate records were removed and the remaining articles were screened by title, abstract and assessed for eligibility by reading the full-text. Finally, we identified six studies that were eligible for review from three countries: India, Phillippines, the Democratic Republic of Congo and Brazil.

### Definitions

Lara and Nolasco [10] differentiated between three categories of self-healing: early healing, late healing and complete healing. Early healing indicated that “all active lesions must have subsided for about six months or longer, within three years after the appearance of the initial lesion or lesions”, while late healing considered “healing which occurs after three years from the appearance of the initial lesion or lesions”. Complete healing was described to be reached when all lesions have disappeared, “leaving no trace whatsoever, or only ordinary looking scars which may be non anaesthetic or partially/totally anaesthetic (with or without a halo or fringe of anaesthesia) [10]. Ekambaram and Sithambaram [8] listed a number of factors to be met in order to determine the occurrence of self-healing, such as “no development of new lesions, absence of old lesions without residual infiltration, absence of neuritis, absence of increase or decrease of anaesthesia or development of any deformity, bacteriologically negative from multiple sites” [8]. Finally, Browne [7] considered self-healing in the case of “progressive repigmentation and resolution in the absence of any systemic anti-leprosy treatment, or local physical treatment received from native healers”. The three remaining studies did not provide a definition, which is consistent with the absence of a consensus definition [7]. In terms of defining healing time of lesions, one study defines it as the “time interval between disease onset and its inactivation or subsidence” [20].

We used the following definition of self-healing, based on the definition by Browne [7]: “Self-healing in leprosy is the disappearance of signs of active infection and/or the complete or partial resolution of lesions, with no redness in lesions or progression in size and or number, and disappearance of any other signs of the disease, including nerve involvement and/or any bacilli in a skin smear, for at least one year without administering anti-leprosy treatment, including MDT [7].”

### Geographic and demographic factors

The studies included present findings from the period between 1938–1978. The data are from six studies that were conducted in four countries of three continents: India and the Philippines (Asia), the Democratic Republic of Congo (Africa), and Brazil (South America).

The two studies from India were conducted in high-endemic leprosy settings, involving persons affected by leprosy through case detection via annual surveys [20,8]. While Sirumban et al. [20] observed 117 untreated PB-cases over a period of 12 years, Ekambaran et al. [8] collected findings from 432 (mostly) tuberculoid and a few “maculo-anaesthetic” leprosy cases. According to Sirumban et al. [20] 22.4% of the PB-cases self-healed. The authors stated that 39% of those healed within a period of two years [8, 20].

Among the tuberculoid and as described by the authors as “maculo-anaesthetic” (probably borderline-tuberculoid) cases, it was reported that 74% self-healed, (86% among these had single lesions) [8]. Furthermore, differences were found between age, nerve involvement, and appearance of single/multiple patches. Thus, cases in the age groups between 0–14 and 15–24 years showed the highest percentage in self-healing. Of those with nerve involvement and single patches, 19.6% self-healed, while only 8.9% with multiple patches, and 4.4% of cases with both nerve involvement and multiple patches self-healed. The third Indian study found similar differences between leprosy types and their probability to self-heal. Ramanujam [21] observed high proportions of self-healing in children over a period of 19 years, differing between children with major tuberculoid, (99% self-healed), minor tuberculoid (78% self-healed) and maculo-anaesthetic leprosy lesions (55% self-healed). No further information was given about the study sample and circumstances [21].

The study by Lara and Nolasco [10] specifically looked at the occurrence of self-healing in children born on the Philippine Island of Culion, which is known as a former leprosy colony due to its sanatorium. As mothers were often suffering from leprosy, children contracted the disease soon after birth, with the first symptoms appearing from six months to a few years after being born. In the period between 1932 and 1956, 287 out of 347 children self-healed from leprosy. The examinations included skin smear-tests, different types of initial lesions and tests of Mitsuda reactions [10]. This study indicated that all forms of leprosy showed a trend towards self-healing, with 62% of 178 cases healing early, and 15.7% late. Nonetheless, differences could be identified. “The papulonodular forms and other definitely circumscribed, thickened lesions were associated with the highest proportions of subsequently healed cases, the infiltration-like, more or less diffusely thickened lesions, with the lowest proportion [21]”.

The study by Browne [7] was conducted in the Democratic Republic of Congo and included 45,035 people of Bantu origin. To obtain all records from people affected by leprosy, 18 health centers and 46 treatment centers were involved, conducting annual whole-population surveys. Out of the complete study population, 2,749 patients were observed with self-healing lesions with a higher incidence in men (n = 1,630) than in women (n = 1,119). Those 2,749 also included patients that may have had lesions for many years prior to the eight-year observer period [7]. Thus, to determine the true incidence of new leprosy cases, Browne [7] focused on all cases diagnosed within the last two years of the eight-year study period. Out of a total of 673 newly diagnosed patients, a third had presented with self-healing lesions during the two-year period. Patients were categorized as “self-healed” or “spontaneously regressed”, if they appeared with progressive repigmentation and/or resolution, without administering anti-leprosy treatment or local treatment by native healers [7].

The last study was conducted in Brazil by Fakhouri et al. [9] and consisted of three study groups, composed of children and adults. The first group consisted of 11 children with nodular leprosy, composed of three boys and eight girls. The tuberculoid group comprised 23 children, with nine men and 14 women. The third group was composed of 24 adults, with 11 men and 13 women. All patients presented with a positive Mitsuda test, indicating the ability of the cell-mediated immune system to respond to the *M*. *leprae* infection. The study showed that all children with nodular leprosy healed completely. Fakhouri et al. [9] assumed that nodular leprosy manifests as a congenital immunity and allergy to *M*. *leprae*. Fakhouri et al. [9] further suspected that the entry point of the infection is a decisive indicator for the person’s immune response and potential self-healing. However, this study did not present any conclusive findings regarding this assumption [9].

Three of the included studies reported that the average healing time was two years [10,20,7]. Lara and Nolasco [10] indicated a longer healing period of 3.25 years for those with tuberculoid lesions; two studies found that healing time was not influenced by number and site of leprosy lesions [10,20,7]. Along with that, it was observed that age, sex, and the intrafamilial leprosy contact status did not affect the time of self-healing [20,7].

## Discussion

We included six studies conducted between 1938 to 1978 exploring the incidence of self-healing in different countries and continents. Children and paucibacillary (PB) casesshowed a high probability of self-healing with an average healing time of two years.

The six studies provided an overview of the current state of knowledge regarding self-healing in leprosy cases. It was obvious that the evidence of self-healing in leprosy patients is scarce and that the studies are more than 45 years old [7,8,9,10]. This can be attributed to the fact that it is ethically problematic not to provide immediate treatment after the leprosy diagnosis has been confirmed, making it difficult to study the natural history of leprosy and thus to determine an accurate incidence of self-healing cases [22].

It may also relate to the lack of an agreed definition of self-healing. We propose the following definition, modifying the one proposed by Browne (1974) [7].

*“Self-healing in leprosy is the disappearance of signs of active infection and/or the complete or partial resolution of lesions*, *with no redness in lesions or progression in size and or number*, *and disappearance of any other signs of the disease*, *including nerve involvement and/or any bacilli in a skin smear*, *for at least one year without administering anti-leprosy treatment*, *including MDT* [7].*”*

Following this definition, self-healing is probably a relatively common phenomenon where the body’s immune system combats the causative agent, *M*. *leprae*, resulting in disappearance of the infection and remission of any signs or symptoms of disease without treatment or other medical intervention. Many studies have described evidence of infection in the form of seropositivity of antibodies to the *M*. *leprae* specific antigen PGL-1. In high endemic areas, rates of seropositivity of 30–40% have been described [23]. Yet, of these only a small minority go on to develop signs of leprosy, showing that the body was capable of resolving infection: preventing disease and clearing the infection without treatment [23].

Although self-healing appears to be described rarely, understanding this process and integrating it into research, might offer perspectives and inform potential avenues for future interventions. It would also add precision to mathematical models predicting the impact of interventions for the prevention of leprosy. Furthermore, awareness and knowledge about self-healing could help estimate the ramifications of phenomena such as the pandemic on untreated leprosy patient cohorts. With that, gaining insights from healthcare professionals and communities about the occurrence of self-healing may be beneficial for monitoring purposes.

In the literature reviewed, self-healing appears to be associated with the type of leprosy and age. While it is difficult to find a definite pattern due to the limited number of studies, most results point to an increased probability of self-healing among children and PB cases [7,8,9,10]. The suggested healing time of two years seems to be consistent in the three studies [10,20,7]. Furthermore, it is biologically plausible that a proportion of patients would self-heal in the absence of treatment. The body’s immune system can kill and clear a wide variety of pathogens, including *M*. *leprae*. It has been estimated that over 95% of individuals show resistance to leprosy. After exposure protection is believed to occur promptly, often without resulting in any visible signs of the disease [24]. PB cases are known to have a cell-mediated immune system capable of mounting a targeted attack on *M*. *leprae*. So, even if the innate immune system does not kill the bacteria at an early stage of the infection, persons with either sub-clinical disease or overt PB disease are likely to have the capacity to resolving the infection. In children, the bacterial load is often small, which may be a reason why they have an increased tendency to resolving the infection.

The phenomenon of self-healing also has operational implications for contact screening and control measures under low-endemic circumstances. Contact screening aims to find new cases among contacts of recently detected new cases. However, if these have minor symptoms and most likely PB disease, missing an occasional case is unlikely to have major consequences regarding *M*. *leprae* transmission and disease impact. Also, persons without clear signs of leprosy can thus be safely kept on observation. The exception to this is early Multibacillary (MB) disease, which may also present without anesthetic skin lesions or clearly enlarged nerves. A skin smear, quantitative Polymerase Chain Reaction (qPCR) test on a skin biopsy, or PGL-1 blood test may confirm that the person in fact has MB leprosy and should be treated without delay [23].

### Strenghts and limitations

This systematic review was the first to examine the phenomenon of self-healing in leprosy, identifying the available evidence of self-healing in leprosy in the published literature. The evidence is intriguing, but is based on relatively few and mostly older studies limited to the English language. While the scientific rigor of these late studies does not entirely meet contemporary publication standards, the data they provide remain valuable for informing current leprosy strategies today. In addition, the included studies provide geographical variation.

This review highlighted the importance of collecting additional data on self-healing among leprosy patients. For example, carefully documenting instances where patients were diagnosed but lacked treatment due to the unavailability of MDT during the pandemic or other barriers in the supply chain. MDT has been provided free of charge through WHO since 1995, but shortages at various (sub-) national levels are occasionally experienced [4,25,26]. Monitoring and analysis of such cases may yield additional insights into the phenomenon of self-healing among leprosy patients.

## Conclusion and recommendations

Recent research on self-healing in leprosy is scarce and evidence limited. This is primarily due to ethical concerns regarding withholding effective treatment of diagnosed patients, and because of the absence of an agreed definition of self-healing. Nevertheless, self-healing appears to be a plausible phenomenon influenced by demographic factors, and the type of leprosy. There is no evidence that the rate of self-healing is impacted by geographical factors. However it is likely that the rate of self-healing is influenced by environmental and socio-economic factors, including poverty, since they affect the the immune system. The status of the latter is likely to play an important role in self-healing. We recommend further research on self-healing in leprosy if and when situations occur that patients have been unable to obtain treatment due to external reasons, as it provides insight into the human immune system and the determinants of this phenomenon.

## Supporting information

S1 Table Search strategy. (DOCX)

S2 Table Study characteristics. (DOCX)

S1 PRISMA Checklist Item checklist. (DOCX)
