# Supplementary material for: Self-healing in leprosy: A systematic review
Source: PLoS Negl Trop Dis. 2024 Sep 12;18(9):e0012434. doi: 10.1371/journal.pntd.0012434 (PMC11392240; doi:10.1371/journal.pntd.0012434)
Supplement: S1 Table — (DOCX) [file pntd.0012434.s001.docx]

# **SI 1** Table 1 Search Strategy

PubMed (27.09.2023)

| Number | Search | Results |
| --- | --- | --- |
| #1 | "leprosy"[MeSH Terms] OR "hansen disease"[Title/Abstract] | 23,319 |
| #2 | "spontaneous*" AND "heal*"[Title/Abstract] OR "self heal*"[Title/Abstract] OR "remission" | 222,490 |
| #3 | #1 AND #2 | 95 |

Web of Science (28.08.2023)

| Number | Search | Results |
| --- | --- | --- |
| #1 | (TS=(leprosy)) OR TS=(hansen disease) | 13,149 |
| #2 | (((TS=(spontaneous*)) AND TS=(heal*)) OR TS=(self-heal*)) OR TS=(remission) | 196,068 |
| #3 | #1 AND #2 | 76 |

Infolep (28.08.2023)

| Number | Search | Results |
| --- | --- | --- |
| #1 | Leprosy | 111 |
| #2 | Self healing | 156 |
| #3 | #1 AND #2 | 156 |

# 
