# Supplementary material for: Self-healing in leprosy: A systematic review
Source: PLoS Negl Trop Dis. 2024 Sep 12;18(9):e0012434. doi: 10.1371/journal.pntd.0012434 (PMC11392240; doi:10.1371/journal.pntd.0012434)
Supplement: S2 Table — (DOCX) [file pntd.0012434.s002.docx]

# **SI 2** Table 2 Study Characteristics

| Author, Year | Title | Country | Study Design | Study Population & Circumstances | Definition of Self-Healing | Results |
| --- | --- | --- | --- | --- | --- | --- |
| Ekambaram, V., Sithambaram, M. (1977) [1] | Self-Healing in non-lepromatous leprosy in the area of the ELEP leprosy control project Dharmapuri (Tamil Nadu) | India | Observational study | - Patients diagnosed with non-lepromatous type or intermediate group of leprosy (detected by survey/ other methods) who never took any treatment in the 6-year period (1.1.1970-31.12.1975) - out of 714 patients, 432 patients (60%) were available for a clinical and bacteriological examination after 6-year period - 425 cases consist mostly of Tuberculoid and a few Maculo-anaesthetic cases | - development of no new lesions - absence of old lesions without residual infiltration - absence of neuritis - absence of increase or decrease of anaesthesia or development of any deformity - bacteriologically negative from multiple sites | - out of 432 cases 315 cases/72.92% self-healed - 90 cases/20.83% remained stationary - 27 cases/6.25% became worse - among 425 Tuberculoid & a few Maculo-anaesthetic cases, 73.8% self-healed, among those 86.2% with single lesions - highest percentage in self-healing is in the age group 0-14 and 15-24. - 66.67% of patients with single patches & without nerve involvement self-healed - 19.61% of those with single patches and nerve involvement healed. - 8.89% of those with multiple patches & no nerve involvement healed. - 4.4% of those with multiple patches and nerve involvement healed |
| Lara, C.B., Nolasco, J.O. (1956) [2] | Self-Healing, or abortive and residual forms of childhood leprosy and their probable significance | Philippines | clinical trial with control group | - verified and probable cases of leprosy found through frequent examinations of all Culion-born children - those nonleprous from outside with their leprous mothers - since 1932, who have been studied for >2 years following the onset of the disease up to March 1956. - in total: 287/ out of 347 cases detected during the 24-year study period - bacteriological examinations include smears from scraping, mitsuda reaction, type of initial lesions, subsequent course. - 104 children receiving repeated intracutaneous lipromin injections (3 injections at 4 months intervals) before appearance of leprous lesions observed over 10 years - 105 children in control group | - **Complete healing:** Disappearance of all lesions leaving no trace whatsoever, or only ordinary-looking scars which may be non- anesthetic or partially or totally anesthetic, with or without a halo or fringe of anesthesia. - Anesthetic scars, either depressed or with crushed-tissue-paper surface, are indicative of former tuberculosis lesions. - **Early healing:** All active lesions must have subsided for about six months or longer, within three years after the appearance of the initial lesion or lesions - **Late healing:** Healing which occurs after three years from the appearance of the initial lesion or lesions. | - Apparent that all forms showed distinct trend toward healing - highest proportion of healed cases: those with thickened & well-circumscribed lesions - lowest proportion: those who had diffuse infiltrations - most benign: papulonodular, discrete lichenoid, indurated, scar-like lesions - followed by wheal-like forms (irregular in contour), raised & flat macules. - complete healing: 77.7% of all cases, - incomplete healing: 12.5%, - trend to progress: 9.7%. - the tendency to persist & advance in the different forms was in reverse order to their tendency to heal - it is not improbable that complete healing may occur in ca. 80% of all childhood infections - shortest healing time: cases with the lichenoid, scar-like, and papulonodular lesions - equal: those with wheal-like lesions, raised macules - longest to heal: flat macules. - average healing time for cases with undifferentiated histology: 2 years - for those with tuberculoid lesions: 3.25 years - Of the total of 223 healed cases, 7.2% suffered true relapse. - Relapse after apparent healing more common among those with lesions of tuberculoid structure, than those with lesions of undifferentiated nature (ratio 3:1) - relapse ratio of clinical form of initial lesions: 1:25 papulonodular, 1:10 raised macules, 1:8 wheal-like & flat macular lesions - no case of relapse: healed cases with scar-like indurated/ lichenoid early lesions. - most relapses occurred within 3 years after apparent self-healing, second healing was mostly noted after 5 years from appearance of initial lesions - group of children receiving lepromin injections before appearance of leprous lesions showed self-healing in 97.5% of cases after 10 years - control group: 80% healed - suggestion that lepromin (Mitsuda) testing may have protective value when giving to young children, evidence however inconclusive |
| Browne, S.G. (1974) [3] | Self-Healing Leprosy: Reports on 2749 Patients | Democratic Republic of Congo | Observational study | - 45,035 persons of Bantu origin representing several tribes living in the medical sector of the Baptist Missionary Society, Yakusu, small villages along the river Congo and in the equatorial rain forest - served by 18 health centers & 36 treatment centers - complete records of all persons suffering from leprosy were obtained through annual whole-population surveys, 6 skin smears were performed on every patient - population was very leprosy-conscious - 2749 patients observed with self-healing lesions - 1630 males, 1119 females. - under observation for 8 years, this includes patients discovered during the initial survey who may have had lesions for several years - during the last 2-year period, the actual incidence of cases of leprosy newly arising could be accurately determined (includes 673 patients) | lesions were included in the category of self-healing or spontaneous regression if at successive examinations they showed progressive repigmentation and resolution, in the absence of any systemic anti-leprosy treatment (prescribed, or clandestine) or local physical treatment (burning, cutting, scarifying etc.) at the hands of native "healers" | - 2749 patients with indeterminate or tuberculoid leprosy observed with self-healing lesions - 1630 males, 1119 females - cases who self-healed: 220 cases (out of 673) - Percentage of leprosy patients with self-healing lesions: 33% - unknown proportion of those suffering from abacillary or paucibacillary forms of leprosy, diagnosed clinically as indeterminate" or "tuberculoid" had lesions that might have proved to regress spontaneously had they not been given treatment - commonest type of regression occurs when the edge becomes sharp defined ant the skin in the macule becomes thicker |
| Fakhouri,R., Sotto, M. N., Manini, M.P.I., Margarido, L.C. (1938) [4] | Nodular (tuberculoid) leprosy of childhood and tuberculoid leprosy: a comparative, morphologic, immunopathologic and quantitative study of skin tissue reaction | Brazil | comparative, morphologic, immunopathologic and quantitative study of skin tissue reaction | - Skin biopsies were obtained from 11 patients with clinical diagnosis of NL, and from 23 children and 24 adult patients with classical tuberculoid leprosy. - 11 infants and children (3 males and 8 females) constituted the NL group (group 1), with ages ranging from 19 months to 9 yrs (mean of 3.61 yrs). - The tuberculoid leprosy children group (group 2) was composed of 23 patients (9 males and 14 females), with ages ranging from 2 to 14 years of age (mean of 8.63 yrs). - The adult group (group 3) included 24 patients (11 male and 13 female) with ages ranging from 19 to 69 yrs (mean of 40.33 yrs). - The Mitsuda skin test was positive in all patients. - 9 NL patients were born to lepromatous mothers, and the remaining had close relatives (one father and one aunt) with lepromatous disease. - complete clinical records and follow up (mean duration of 5.9 yrs) were accessed, and all patients presented an auto-resolutive course of disease without specific treatment. - Only one patient received chaulmoogra oil treatment. - All of them resolved without progression to other disease forms or deformities. | none provided | - Group 1: All patients with nodular lesions resolved to healing without disease progression or deformities. - In spite of neural damage seen in skin lesions, patients with nodular leprosy resolve without peripheral nervous system injury such as neuritis, amyotrophy, paralysis, or trophic lesions. - We observed AFB in 18.18% of NL biopsies and Mycobacterium antigen in 72.72% of the specimens. |
| Sirumban, P., Kumar, A., Neelan, P.N. (1988) [5] | Healing time in untreated paucibacillary leprosy: a cross-sectional study | India | cross-sectional survey study | - Where: Rural Field Operation Area of the Central Leprosy Teaching and Research Insititute (Tamil Nadu) - Population of 95,000 persons residing in 54 villages - study period of 12 years (1969-1980) - annual detection surveys + clinical & bacteriological examination - Study Population: all patients were advised to take treatment, but most PB-type cases did not take treatment, the progress of those 117 participants was recorded during annual surveys & further examination | healing time of a lesion may be defined as the time interval between disease onset (overt disease) and its inactivation or subsidence. | - Healing time of leprosy lesions in 117 untreated (PB) cases: estimated to be 2.03 ± 0.10 (mean ± S.E.) years - This healing time does not appear to be influenced by epidemiological characteristics of the patients such as age, sex, intrafamilial leprosy contact status, number and site of leprosy lesions, etc. - the rate of healing and downgrading among the total untreated cases was 22.4% and 0.57%, respectively, per year - About 39% of the total untreated PB leprosy cases healed within a period of 2 years. |
| Ramanujam, K. (1978) [6] | The Saidapet story, findings of a nineteen-year follow-up of children with untreated leprosy | India | Observational study |  | none provided | - observed a high proportion of spontaneous healing in children with major tuberculoid (98.7%), minor tuberculoid (78%), and maculo anesthetic leprosy lesions (55%) |

**References**

1. Ekambaram V, Sithambaram M. Self-healing in non-lepromatous leprosy in the area of the ELEP Leprosy Control Project Dharmapuri (Tamil Nadu). Lepr India. 1977;49(3):387-392.
2. Lara CB, Nolasco JO. Self-healing, or abortive, and residual forms of childhood leprosy and their probable significance. Int J Lepr. 1956;24(3):245-263.
3. Browne SG. Self-healing Leprosy: Report on 2749 Patients. Lepr Rev. 1974;45:104-111.
4. Fakhouri R, Sotto MN, Manono MPI, Margarido LC. Nodular leprosy of childhood and tuberculoid leprosy: a comparative, morphologic, immunopathologic and quantitative study of skin tissue reaction. Int J Lepr Other Mycobact Dis. 1938;71(3).
5. Sirumban P, Kumar A, Neelan PN. Healing Time in Untreated Paucibacillary Leprosy: A Cross-sectional Study. Int J Lepr Other Mycobact Dis. 1988;56(2).
6. Ramanujam K. The Saidapet story, findings of a nineteen year followup of children with untreated leprosy. (Abstract). Int J Lepr. 1979;47:347-348.
